# Respective Contributions of URT1 and HESO1 to the Uridylation of 5′ Fragments Produced From RISC-Cleaved mRNAs

**DOI:** 10.3389/fpls.2018.01438

**Published:** 2018-10-09

**Authors:** Hélène Zuber, Hélène Scheer, Anne-Caroline Joly, Dominique Gagliardi

**Affiliations:** Institut de Biologie Moléculaire des Plantes (IBMP), Centre National de la Recherche Scientifique (CNRS), Université de Strasbourg, Strasbourg, France

**Keywords:** uridylation, TUTase, RISC, RNA silencing, Arabidopsis, RNA degradation, miRNA, Illumina

## Abstract

In plants, post-transcriptional gene silencing (PTGS) represses gene expression by translation inhibition and cleavage of target mRNAs. The slicing activity is provided by argonaute 1 (AGO1), and the cleavage site is determined by sequence complementarity between the target mRNA and the microRNA (miRNA) or short interfering RNA (siRNA) loaded onto AGO1, to form the core of the RNA induced silencing complex (RISC). Following cleavage, the resulting 5′ fragment is modified at its 3′ end by the untemplated addition of uridines. Uridylation is proposed to facilitate RISC recycling and the degradation of the RISC 5′-cleavage fragment. Here, we detail a 3′ RACE-seq method to analyze the 3′ ends of 5′ fragments produced from RISC-cleaved transcripts. The protocol is based on the ligation of a primer at the 3′ end of RNA, followed by cDNA synthesis and the subsequent targeted amplification by PCR to generate amplicon libraries suitable for Illumina sequencing. A detailed data processing pipeline is provided to analyze nibbling and tailing at high resolution. Using this method, we compared the tailing and nibbling patterns of RISC-cleaved *MYB33* and *SPL13* transcripts between wild-type plants and mutant plants depleted for the terminal uridylyltransferases (TUTases) HESO1 and URT1. Our data reveal the respective contributions of HESO and URT1 in the uridylation of RISC-cleaved *MYB33* and SPL13 transcripts, with HESO1 being the major TUTase involved in uridylating these fragments. Because of its depth, the 3′ RACE-seq method shows at high resolution that these RISC-generated 5′ RNA fragments are nibbled by a few nucleotides close to the cleavage site in the absence of uridylation. 3′ RACE-seq is a suitable approach for a reliable comparison of uridylation and nibbling patterns between mutants, a prerequisite to the identification of all factors involved in the clearance of RISC-generated 5′ mRNA fragments.

## Introduction

Small RNAs are key regulators of gene expression (Borges and Martienssen, 2015; Bartel, 2018). They are classified as two main types, microRNAs (miRNAs) and short interfering RNAs (siRNAs), because of key distinctions in their respective mode of biogenesis (Martínez de Alba et al., 2013; Borges and Martienssen, 2015). miRNAs are processed from primary transcripts that fold as a hairpin with an imperfectly paired stem. By contrast, siRNAs are generated from near-perfect double stranded RNAs (dsRNAs) or fully paired dsRNAs when the complementary strand is synthesized by a RNA-dependent RNA polymerase (RDR), which uses the sense strand as template. miRNAs and siRNAs are loaded onto members of the argonaute (AGO) protein family to form the core of RNA induced silencing complexes (RISCs) (Vaucheret, 2008; Zhang et al., 2015). RISCs are then guided to their targets by sequence complementarity with the loaded small RNA. In plants, the base pairing of miRNAs with their targets is rather extensive, and mRNAs regulated by RISCs are repressed by AGO1-mediated cleavage, but also by translation repression (Chen, 2004; Brodersen et al., 2008; Yang et al., 2012; Li et al., 2013; Iwakawa and Tomari, 2015; Reis et al., 2015; Arribas-Hernández et al., 2016). Cleavage of mRNAs by RISC produces a 5′ fragment and a 3′ fragment. As detailed below, both the 5′-3′ and 3′-5′ RNA degradation pathways contribute to the elimination of these fragments.

In Arabidopsis, the cytosolic 5′-3′ exoribonuclease XRN4 participates in the degradation of RISC 3′-cleavage fragments, as indicated by their accumulation in *xrn4* mutants (Souret et al., 2004). XRN4 was also proposed to be involved in the degradation of RISC 5′-cleavage fragments because the 5′ fragment resulting from the cleavage of *MYB domain protein 33* (*MYB33*) mRNA by miR159-loaded RISC accumulates in a *xrn4* mutant (Ren et al., 2014). RISC 5′-cleavage fragments are also definitely degraded by the 3′-5′ RNA degradation pathway because they accumulate in *ski2, ski3*, and *ski8* mutants (Branscheid et al., 2015). Together, SKI2-3-8 form the Ski complex, which is the major activator of the RNA exosome in the cytosol. Therefore, the involvement of the RNA exosome in the degradation of RISC 5′-cleavage fragments is likely in Arabidopsis, although it remains to be demonstrated using appropriate mutants. This implication of the RNA exosome would be consistent with previous findings in other organisms, such as *Drosophila melanogaster* (Orban and Izaurralde, 2005). In addition, two ribonucleases were recently described in Arabidopsis as taking part in the metabolism of RISC 5′-cleavage fragments: RISC-interacting clearing 3′-5′ exoribonucleases 1 and 2 (RICE-1 and -2) (Zhang et al., 2017). RICEs are homohexamers with a DnaQ-like exonuclease fold, and they interact with AGO1 and AGO10 (Zhang et al., 2017). RICEs are proposed to initiate the destabilization of RISC 5′-cleavage fragments thereby facilitating RISC dissociation. This would grant access of the 3′ extremity of RISC 5′-cleavage fragments to the RNA exosome and importantly, recycle RISC, which is essential to maintain functional RISC and miRNA abundance (Zhang et al., 2017). The access of the 3′ extremity of RISC 5′-cleavage fragments to the RNA exosome may also be facilitated by components of the non-stop decay (NSD) pathway when the RISC 5′-cleavage fragment is engaged in polysomes (Szádeczky-Kardoss et al., 2018). The prime function of NSD is to eliminate mRNAs lacking stop codons. Recently, orthologs of Pelota and Hbs1, two core components of NSD, were shown to participate in the elimination of RISC 5′-cleavage fragments in *Nicotiana benthamiana* and *A. thaliana*, provided that the cleavage site is within the coding region (Szádeczky-Kardoss et al., 2018). Likely, the NSD machinery promotes the dissociation of ribosomes stalled at the extremity of a RISC 5′-cleavage fragment to promote access to the RNA exosome (Szádeczky-Kardoss et al., 2018).

Besides exoribonucleases and RNA helicases, terminal uridylyltransferases (TUTases) constitute another type of enzymatic activities involved in the clearance of RISC 5′-cleavage fragments. Indeed a striking molecular event in this process is the untemplated addition of uridines at the 3′ extremity of RISC 5′-cleavage fragments (Shen and Goodman, 2004; Ren et al., 2014; Zhang et al., 2017). The uridylation of several of such fragments was originally reported in both Arabidopsis and mice (Shen and Goodman, 2004). Since then, uridylation has emerged as a conserved post-transcriptional process that shapes the coding and non-coding transcriptomes in eukaryotes (Munoz-Tello et al., 2015; Scheer et al., 2016; De Almeida et al., 2018). In Arabidopsis, two TUTases have been characterized: HEN1 SUPPRESSOR 1 (HESO1) and URIDYLYLTRANSFERASE 1 (URT1) (Kwak and Wickens, 2007; Ren et al., 2012; Zhao et al., 2012; Sement et al., 2013). Both HESO1 and URT1 contain the core catalytic domain (CCD) that defines proteins belonging to the terminal nucleotidyltransferase family. In addition, URT1 contains a large intrinsically disordered region (IDR) in its N-terminal region, while a shorter IDR is present in the C-terminal region of HESO1 (De Almeida et al., 2018). Those IDRs may mediate the recognition of protein partners by URT1 and HESO1, or be a key to their localization in P-bodies and/or stress granules (Sement et al., 2013; Ren et al., 2014; Wang et al., 2015). HESO1 was identified as the main TUTase uridylating miRNAs and siRNAs to trigger their degradation (Ren et al., 2012; Zhao et al., 2012). In addition, HESO1 was shown to uridylate three RISC 5′-cleavage fragments (Ren et al., 2014). Those fragments are generated from *MYB33, Auxin Response Factor 10* (*ARF10*), and *Lost Meristems 1* (*LOM1*) mRNAs, which are targets of miR159, miR160, and miR171, respectively. A residual uridylation of these RISC 5′-cleavage fragments is observed in *heso1* mutants (Ren et al., 2014) and, this secondary activity may be due to URT1, although experimental evidence supporting this hypothesis is lacking to date. URT1 is the main TUTase uridylating mRNAs in Arabidopsis, because mRNA uridylation is decreased by 70–80% in *urt1-1* mutants (Sement et al., 2013; Zuber et al., 2016). URT1 can also uridylate miRNAs, mostly when HESO1, the primary TUTase involved in small RNA uridylation, and HUA ENHANCER 1 (HEN1), a methyltransferase that methylate small RNA duplexes, are absent (Yu et al., 2005; Yang et al., 2006; Ren et al., 2012; Zhao et al., 2012; Tu et al., 2015; Wang et al., 2015). miRNAs are therefore the first documented example of shared RNA substrates between HESO1 and URT1 (Tu et al., 2015; Wang et al., 2015). Yet, both overlapping and distinctive roles in miRNA uridylation were attributed to each TUTase (Tu et al., 2015; Wang et al., 2015). mRNAs and RISC-cleaved transcripts could constitute other cases of shared RNA substrates between HESO1 and URT1. These possibilities remain to be experimentally addressed.

To date, the characterization of uridylated RISC 5′-cleavage fragments has relied on the use of 3′ RACE PCR followed by cloning and subsequent analysis based on Sanger sequencing. Although this experimental strategy has proven useful, it has some inherent limitations. The first one is that this method is low-throughput. It is time-consuming and the depth is usually quite limited, with often less than 20–30 clones analyzed per genotype. The second major drawback is the lack of discrimination between amplicons and independent molecules. This turns out to be a real issue when analyzing low complexity samples by PCR amplification, with the majority (up to 90% as determined here during the analysis of RISC 5′-cleavage fragments) of the final PCR products that correspond to very few independent templates. Taken together, these limitations hinder the qualitative and quantitative analysis of the uridylation of RISC-cleaved transcripts. Such an analysis is crucial to reliably compare uridylation between wild-type (WT) and mutant genetic backgrounds, and this comparison is required to identify all factors involved in the metabolism of 5′ RNA fragments produced by RISC cleavage.

Here we detail a 3′ RACE-seq method that has been optimized for analyzing the uridylation of 5′ fragments from RISC-cleaved transcripts. Those molecules are usually low abundant within the complex mixture of all cellular RNAs, and they exhibit a rather poor diversity, with a few untemplated nucleotides usually added at a precise RISC-mediated cleavage site. We illustrate the use of 3′ RACE-seq to analyze the tailing and trimming patterns of *MYB33* and *SPL13* RISC 5′-cleavage fragments by comparing WT plants and mutants lacking HESO1 and URT1. This analysis revealed the respective contributions of both TUTases, and that the absence of uridylation results in the accumulation of 5′-cleavage fragments nibbled by a few nucleotides close to the site cleaved by RISC.

## Materials and Methods

### Gene IDs and Primers

The Arabidopsis Genome Initiative (AGI) locus identifiers for the genes studied in this study are: AT2G39740 (*HESO1*), AT2G45620 (*URT1*), AT5G06100 (*MYB33*), and AT5G50570 (*SPL13A*). Please note that AT5G50570 (*SPL13A*) and AT5G50670 (*SPL13B*) have identical coding sequences and therefore cannot be discriminated in this study. For simplicity, the name *SPL13* is used thereafter. The sequence of all primers used in this study is shown in **Supplementary Table S1**.

### Plant Material

The plant material used for analyzing RISC 5′-cleavage fragments by 3′ RACE-seq corresponds to Arabidopsis plantlets of Col-0 accession grown for 24 days *in vitro* on Murashige and Skoog media with 0.8% agar and 12 h light (22°C)/12 h darkness cycles (18°C). For other analyses, flowers were harvested from Arabidopsis of Col-0 accession and grown on soil with 16 h light/8 h darkness cycles. *urt1-1* (Salk_087647C) and *heso1-1* (GK-367H02-017041) T-DNA mutant lines have been previously described (Zhao et al., 2012; Sement et al., 2013). Double mutants were obtained by down regulating *URT1* by co-suppression in *heso1-1*. For this purpose, *heso1-1* plants were transformed with a construct expressing an inactive version of URT1 fused to YFP, which was fortuitously found to efficiently trigger co-suppression of the endogenous *URT1* gene. The sequence encoding the inactive version of URT1 (URT1^D491/3A^) (Sement et al., 2013) was cloned in the pEarleyGate 104 Gateway plasmid under the control of the cauliflower mosaic virus (CaMV) 35S promoter. Analyses were performed on two biological replicates of three independent *heso1-1 urt1*^SIL^ lines. As a control, we also analyzed two biological replicates of a *urt1*^SIL^ line obtained by co-suppressing *URT1* with a *YFP-URT1* sequence cloned in the pEarleyGate 104 Gateway plasmid.

### Protein Extraction and Western Blot Analysis

Proteins were extracted from flowers of WT, *urt1-1, urt1*^SIL^ and three independent *heso1-1 urt1*^SIL^ lines under denaturing conditions. Proteins were resolved on a 8% SDS-PAGE gel and transferred to an Immobilon-P membrane. Immunoblots were incubated with anti-URT1 antibodies raised in rabbits against the full-length recombinant URT1. Following incubation with horseradish peroxidase-coupled secondary antibodies and Lumi-Light Western Blotting Substrate (Roche), signals were recorded using the Fusion-FX system (Vilber Lourmat).

### RNA Extraction and Northern Blot Analysis

Total RNA was extracted from 24-day-old plantlets and flowers for 3′ RACE-seq and northern blot analyses, respectively, with TRI Reagent^®^ (Molecular Research Center) according to manufacturer’s instructions. RNA was further purified by acid phenol: chloroform: isoamyl alcohol extraction followed by ethanol precipitation. For northern blot analysis of *MYB33* RISC 5′-cleavage fragments, 30 μg total RNAs from WT, *urt1-1* and *heso1-1* mutants were separated on a denaturing formaldehyde 1.5% (w/v) agarose gel and transferred onto a nylon membrane (Hybond™-N+, GE Healthcare Life Sciences™). Following UV-cross-link at 120 mJ/cm^2^ for two times 30 s and incubation for 30 min in PerfectHyb Plus Hybridization buffer (Sigma), the membrane was hybridized overnight at 65°C with a probe that detects the 5′ fragment of *MYB33* RISC-cleaved transcripts. The probe was prepared by PCR amplifying a 219 bp sequence upstream of the RISC cleavage site (**Supplementary Table S1**) and by random primed labeling the PCR product using [α-^32^P]-dCTP and DecaLabel DNA labeling kit (Thermo Scientific). For northern blot analysis of miR159, 10 μg total RNA from WT, *urt1-1*, and *heso1-1* mutants were separated on 17.5% polyacrylamide/7 M urea gels and transferred onto nylon membranes (Hybond™-NX, GE Healthcare Life Sciences™). Following UV-cross-link at 120 mJ/cm^2^ for two times 30 s and incubation for 30 min in PerfectHyb Plus Hybridization buffer (Sigma), membranes were hybridized overnight at 50°C with a 5′ [^32^P]-labeled oligonucleotide to detect miR159 (**Supplementary Table S1**). The probe was labeled using [γ-^32^P] ATP and T4 PNK (NEB) according to manufacturer’s instruction. Radioactive signals were detected by autoradiography and quantified using a Typhoon scanner (GE Healthcare Life Sciences) and Image Gauge software. Plant material used for biological replicates 1 and 2 were common for both northern analyses.

### 3′ RACE-Seq Protocol

**FIGURE 1:**
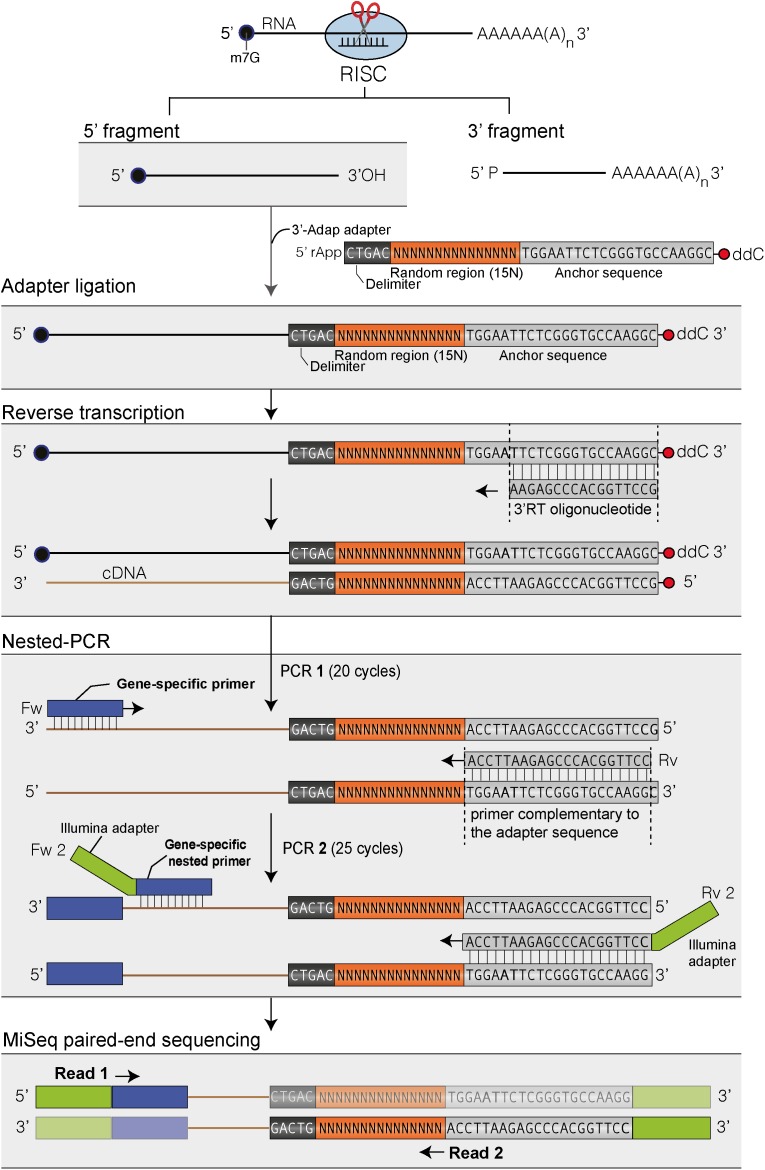
Flowchart of the main steps to map the 3′ ends of RISC 5′-cleavage fragments by 3′ RACE-seq. Features of the 3′ adapter and the principle of the main steps are indicated. The experimental workflow begins by ligating the 3′ adapter to the RISC 5′-cleavage fragment. Please note that any RNA molecule with a 3′ hydroxyl end in the total RNA sample is ligated to the 3′ adapter. The target of interest is specifically amplified during PCR-1 and -2 due to the gene-specific sequences of the forward primers. The protocol is detailed in Methods and the scripts used to analyze data are given in **Supplementary Data Sheets S1, S2**.

A 3′ RACE-seq protocol was adapted for analyzing RISC 5′-cleavage fragments. Total RNA was extracted from 24-day-old seedlings using TRI Reagent^®^ as described above. Twenty pmoles of a 5′-riboadenylated DNA oligonucleotide (3′-Adap, **Figure 1** and **Supplementary Table S1**) were ligated to 10 μg of total RNA using 20 U of T4 ssRNA Ligase 1 (NEB) in a final volume of 100 μl for 1 h at 37°C and 1X T4 of RNA Ligase Reaction Buffer (NEB, 50 mM Tris–HCl, 10 mM MgCl2, 1 mM DTT, pH 7.5). The ligation products were purified from reagents and non-ligated adapter molecules with Nucleospin^®^ RNA Clean-up columns (Macherey Nagel). RNA was then precipitated with ethanol, solubilized in water and quantified. cDNA synthesis was performed in two 20 μl-reactions for each sample. Each 20 μl-reaction contained 2 μg of purified ligated RNA, 50 pmol of the 3′-RT oligonucleotide (**Supplementary Table S1**), 10 nmol of dNTP, 0.1 μmol of DTT, 40 U of RNaseOUT (Invitrogen™), 200 U of SuperScript IV reverse transcriptase (Invitrogen™) and 1X of SuperScript IV RT buffer (Invitrogen™). Reactions were incubated at 50°C for 10 min, and then at 80°C for 10 min to inactivate the reverse transcriptase. The two 20 μl-reactions for each sample were pooled, the cDNAs were extracted with phenol–chloroform, precipitated with ethanol and dissolved in 8 μl Milli-Q water. Two nested PCR amplification rounds of 20 and 25 cycles, respectively, were then performed. PCR1 was run using cDNA synthesized from 1 μg of total RNA, i.e., 2 μl of concentrated cDNA, 10 pmol of *MYB33* or *SPL13* gene-specific sense primer 1, 10 pmol of RACEseq_rev1 primer (**Supplementary Table S1**), 10 nmol of dNTP, 1 U of GoTaq^®^ DNA Polymerase (Promega) and 1X of Green GoTaq^®^ Reaction Buffer (Promega) in a 20 μl final volume. The conditions for PCR1 were as follows: a step at 94°C for 30 s; 20 cycles at 94°C for 20 s, 50°C for 20 s and 72°C for 30 s; a final step at 72°C for 30 s. PCR2 was performed using 1 μl of PCR1 product, 10 pmol of *MYB33* or *SPL13* gene-specific sense primer 2, 10 pmol of a TruSeq RNA PCR index (RPI, **Supplementary Table S1**) 10 nmol of dNTP, 1 U of GoTaq^®^ DNA Polymerase (Promega) and 1X of Green GoTaq^®^ Reaction Buffer (Promega) in a 20 μl final volume. The conditions for PCR2 were as follows: a step at 94°C for 1 min; 25 cycles at 94°C for 30 s, 56°C for 20 s and 72°C for 30 s; a final step at 72°C for 30 s. For each sample, three to four 20 μl-reactions were run and pooled. All PCR2 products were purified using 1 volume of AMPure XP beads (Agencourt). Library concentrations were determined using a Qubit fluorometer (Invitrogen™). Libraries were analyzed on a 2100 Bioanalyzer system (Agilent) to assess quality and estimate size distribution. Library were paired-end sequenced with MiSeq (v3 chemistry) with 41 × 111 bp cycle settings. The respective numbers of sequencing cycles for read 1 and read 2 can be adjusted according to other samples that are co-analyzed. Read 1 is used to identify target transcript whereas read 2 is used to map 3′ extremities and analyze 3′ potential untemplated nucleotides. To compensate for the poor diversity of the amplicon libraries, 25–33% of phiX control library (Illumina) were included. Two rounds of RACE-seq experiments were performed. For the first round, four independent biological replicates were analyzed for WT and *heso1-1* genotypes. For the second round, two independent biological replicates were analyzed for WT, *urt1-1, heso1-1, urt1*^SIL^ line, and each of the three *heso1-1 urt1*^SIL^ lines (i.e., six *heso1-1 urt1*^SIL^ samples). Plant material used for biological replicates 1 and 2 was common for both rounds.

### Bioinformatic Analysis of 3′ RACE-Seq Data

**FIGURE 2:**
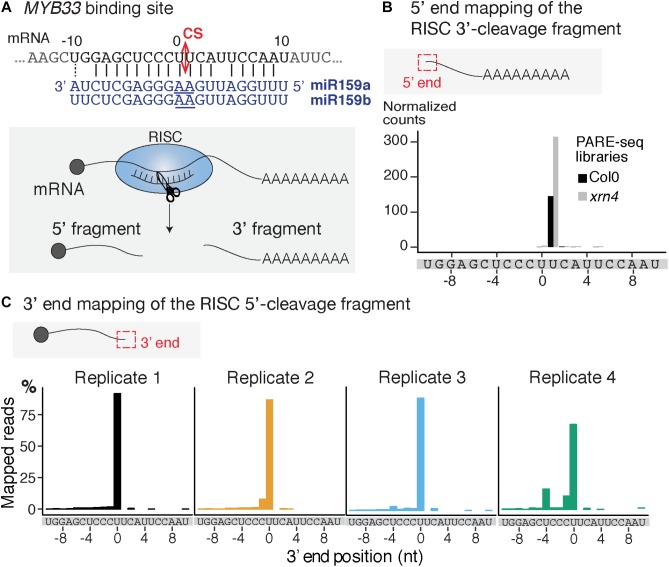
Mapping of the 3′ extremity of *MYB33* RISC 5′-cleavage fragments. **(A)** Schematic representation of the cleavage of *MYB33* mRNA by AGO1 loaded with miR159. The cleavage site (CS) is predicted between the positions 0 and +1, and is defined by the 10th and 11th nucleotides (underlined) of miR159. **(B)** 5′ end mapping using PARE-seq data (German et al., 2008) of RISC 3′-cleavage fragments in WT and *xrn4*. **(C)** 3′ end mapping by 3′ RACE-seq of RISC 5′-cleavage fragments in WT within the –10/ + 10 window corresponding to the *MYB33* sequence recognized by miR159. For 3′ RACE-seq, most reads map to a single position in each of the four WT biological replicates.

Sequencing run quality fit Illumina specification with more than 90% bases higher than Q30. After initial data processing by the MiSeq Control Software v 2.5 (Illumina), base calls were retrieved and further analyzed by a suite of homemade scripts (**Supplementary Data Sheets S1, S2**) using python (v2.7), biopython (v1.63), and regex (v2.4) libraries. Data processing pipeline was adapted from Sikorska et al. (2017). Reads with low quality bases (= < Q10) within the 15-base random sequence of the read 2 or within the 20 bases downstream the delimiter sequence, were filtered out. Sequences with identical nucleotides in 15-base random sequence were deduplicated. Next, the sequences AAGAATTCTCGTCGCCTGAA and GCCAGAGCTATGTTGTTGGT were searched into reads 1 to identify *MYB33* and *SPL13* corresponding reads, respectively. One mismatch was tolerated. Matched reads 1 and their corresponding reads 2 were extracted and annotated. Reads 2 that contain the delimiter sequence were selected and subsequently trimmed from their random and delimiter sequences. In order to map 3′ extremities of target 5′ RISC generated fragments, the 20 nucleotide sequences downstream the read 2 delimiter sequence were mapped to the corresponding reference sequence, which goes from the first nucleotide of the transcript that maps the forward PCR primer MYB33_RISC_fw2 or SPL13_RISC_fw2 to the last nucleotide of the miRNA binding site. To map the 3′ end position of reads 2 with untemplated tails, the sequences of the unmatched reads 2 were successively trimmed from their 3′ end, with a 1 nt trimming step, until successfully mapped to the reference sequence or until a maximum of 30 nt has been removed. For each successfully mapped read 2, untemplated nucleotides at the 3′ end were extracted and analyzed for their size and composition. 3′ modifications longer than 1 were considered only if composed of at least of 50 % of the same base (i.e., 50% U, 50% A, 50% C, or 50% G). As explained in the Results section and as illustrated in **Figures 2, 7** for *MYB33* and *SPL13*, respectively, the sites cleaved by RISC were defined by using PARE-seq datasets to map the 5′ most nucleotide of the 3′ fragment. A single cleavage site was determined for *MYB33*, i.e., between nucleotides at position 0 and position +1, in contrast to two cleavage sites for *SPL13*, i.e., a major site between 0 and +1 and a minor site between +1 and +2. Because position +1 in *SPL13* is a uridine, we could not determine whether this U is encoded or added post-transcriptionally and SPL13 graphs were generated by considering only U-tails > 2. Finally, a supplemental deduplication was performed to increase stringency: sequences with 13 or more identical nucleotides in the 15-base random sequence were deduplicated. Plotting and quantitative data analysis was performed with R software (v3.3.1) and ggplot2 R (v2.2.1). Percentages of uridylated fragments were plotted for reads with 3′ extremities that map at the cleavage site, with U-tails being defined as tail composed of more than 50% U. Data obtained from the two rounds of RACE-seq experiments, referred to as dataset #1 and dataset #2, have been deposited to the NCBI Gene Expression Omnibus (GEO) database with the accession code GSE115470.

## Results and Discussion

### Workflow for Mapping 3′ Ends of RISC 5′-Cleavage Fragments by 3′ RACE-Seq

The principle of 3′ RACE-seq to analyze the 3′ ends of RISC 5′-cleavage fragments is shown in **Figure 1**. Briefly, a 5′ pre-riboadenylated oligodeoxynucleotide adapter is ligated to the 3′ hydroxyl end of RNA molecules using T4 RNA ligase 1 and total RNA. The sequence of the 3′ adapter is identical to the one previously described for the TAIL-seq procedure (Chang et al., 2014). However, unlike for TAIL-seq, the 3′ adapter does not need to be biotinylated. The sequence features of the adapter are detailed in **Figure 1**. Five nucleotides at the 5′ end of the adapter form a delimiter sequence. All reads that do not contain this delimiter sequence are discarded during the analysis. This ensures that we accurately map the 3′ extremity of a transcript that has been ligated to the 3′ adapter. Untemplated nucleotides are defined as any nucleotides present between the genome-encoded sequence and the delimiter. The delimiter is followed by a random sequence of 15 bases. This random sequence is essential to remove PCR duplicates during the bioinformatic analysis. This deduplication step is crucial when using 3′ RACE-seq to analyze low abundant RNA species with limited complexity, which is typically the case for RISC 5′-cleavage fragments. To further prevent amplicon biases due to the misincorporation of nucleotides in the random sequence during PCR amplification or due to sequencing errors of the random sequence, we enhance the stringency of the deduplication step by not tolerating up to two mismatches within the 15-base random sequence of deduplicated sequences.

The 3′ adapter then contains 22 additional bases, which provide an anchor sequence for cDNA synthesis and subsequent PCR amplification. Importantly, the primer used for cDNA synthesis is complementary to the sequence of the 3′ adapter but stops five bases downstream of the random sequence (**Figure 1**). By using a reverse primer for PCR amplification that extends up to the random sequence, we eliminate the vast majority of cDNAs that are due to priming artifacts and specifically analyze transcripts that have the 3′ adapter ligated at their 3′ ends. This trick greatly enhanced the quality and depth of our libraries. Finally, the 3′ adapter is terminated by a dideoxy-C to prevent self-ligation (**Figure 1**).

cDNAs are then subjected to two successive rounds of PCR amplification. For the first round, the forward primer is a gene-specific primer matching the sequence of a selected mRNA and located ideally about 200–400 nucleotides upstream of the predicted RISC-mediated cleavage site. The reverse primer matches the sequence of the 3′ adapter up to the random sequence (**Figure 1**). As mentioned above, this prevents the amplification of most cDNA priming artifacts. The second round of PCR is performed with a nested forward primer and a bar-coded reverse primer complementary to the anchor sequence (**Figure 1**). Typically, thirty different barcodes can be used to simultaneously analyze different genotypes or replicates. Both forward and reverse primers contain 5′ extensions corresponding to the Illumina sequences that are used for flow cell hybridization and sequencing. The number of PCR cycles must be kept as low as possible for both PCRs and ideally should not exceed 20–25 per PCR. Amplicon libraries are purified using AMPure XP beads, quantified with an Invitrogen Qubit fluorometer and their size distribution is determined with a 2100 Bioanalyzer system (Agilent). Amplicon libraries are then sequenced using MiSeq paired-end sequencing for an average yield per run of 38 million of reads: 19 millions of read 1 and 19 millions of read 2.

### Mapping of the RISC-Cleavage Site in *MYB33* mRNAs by 3′ RACE-Seq

We selected the *MYB33* mRNAs targeted by miR159 as a model substrate to set up the mapping of the 3′ ends of RISC 5′-cleavage fragments by 3′ RACE-seq. *MYB33* has been chosen in several studies to investigate uridylation of RISC 5′-cleavage fragments for two main reasons (Shen and Goodman, 2004; Ren et al., 2014; Zhang et al., 2017). First, *MYB33* RISC 5′-cleavage fragments are detectable by northern blot analysis, and therefore their accumulation can be compared between WT plants and relevant mutants. Second, a high proportion of *MYB33* RISC 5′-cleavage fragments is uridylated in WT plants. This proportion was in fact high enough to allow detection of uridylated *MYB33* RISC 5′-cleavage fragments by sequencing of a limited number of clones (Shen and Goodman, 2004; Ren et al., 2014; Zhang et al., 2017). The high level of uridylation in WT plants is useful to monitor decrease of uridylation in mutants to identify factors that are involved in the metabolism of this RISC 5′-cleavage fragment. However, there is one drawback in choosing *MYB33* to study uridylation: the predicted cleavage site, which is specified by the tenth and eleventh nucleotides of miR159, is situated between two uridines (**Figure 2A**). This can lead to uncertainties as to whether some 3′ terminal uridines are genome-encoded or added post-transcriptionally by TUTases. To solve this issue, we took advantage of previous data generated using parallel analysis of RNA ends (PARE)-seq. PARE-seq is one of the sequencing methods designed to map 5′ hydroxylated end of RNAs and used to map small RNA cleavage sites (German et al., 2008). PARE-seq unambiguously identifies the position defined here as +1 as the 5′ nucleotide of the RISC-generated 3′ fragment of *MYB33* (**Figure 2B**). Therefore, cleavage of *MYB33* by miR159-loaded AGO1 does occur at the canonical site, which we defined here between positions 0 and +1 (**Figure 2A**). This was further experimentally validated in the present study because *MYB33* RISC 5′-cleavage fragments ending at position 0 accumulate in a genetic background abolishing uridylation (detailed later in **Figure 6B**).

To study *MYB33* RISC 5′-cleavage fragments by 3′ RACE-seq, we first analyzed the aerial part of 24-day-old plants grown *in vitro* corresponding to four biological replicates for WT and four biological replicates for the *heso1-1* mutant. We obtained a total of 29,689 reads for WT and 34,096 reads for *heso1-1* (**Supplementary Table S2** and **Supplementary Data Sheet S1**). The WT data were first used to monitor the distribution of 3′ extremities mapped in the sequence to which miR159 binds. The majority of reads (up to 85%) mapped at position 0 (**Figure 2C**). Therefore, we conclude that the 3′ extremities of RISC-cleaved *MYB33* are accurately mapped by 3′ RACE-seq.

### Respective Contributions of HESO1 and URT1 in the Uridylation of *MYB33* 5′-Cleavage Fragments

**FIGURE 3:**
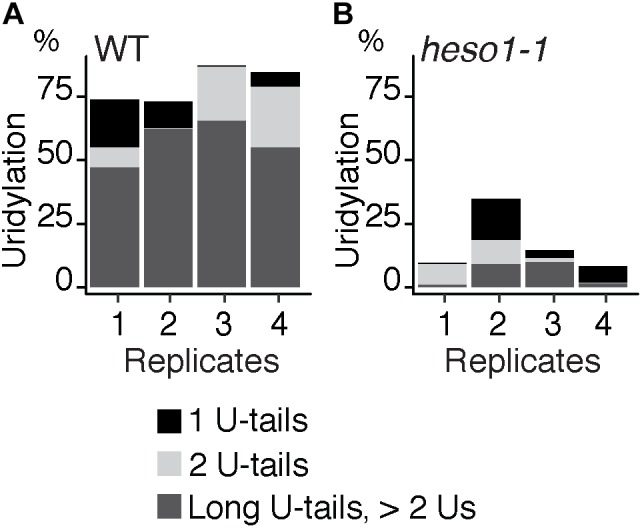
HESO1 is the main TUTase uridylating *MYB33* RISC 5′-cleavage fragments. Percentages of uridylated *MYB33* RISC 5′-cleavage fragments in four biological replicates for **(A)** WT and **(B)** *heso1-1*. Percentages of long (>2 Us), 2 U- and 1 U-tails are indicated by dark gray, light gray, and black, respectively.

To analyze untemplated nucleotides added after RISC-mediated cleavage of *MYB33* mRNAs, the nucleotide extensions for reads that map to position 0 were analyzed first for the WT samples. Up to 98 % of *MYB33* RISC 5′-cleavage fragments in WT are tailed by nucleotide extensions, which are predominantly composed of uridines (**Supplementary Table S3**). This result is in agreement with previous observations (Shen and Goodman, 2004; Ren et al., 2014; Zhang et al., 2017). Most U-rich tails were longer than 2 Us in the four WT biological replicates (**Figure 3A** and **Supplementary Table S3**). We then compared the impact of HESO1 on the uridylation *MYB33* RISC 5′-cleavage fragments. A major decrease in uridylation was observed in *heso1-1* as compared with WT samples (**Figure 3B**). This observation confirmed that HESO1 is the main TUTase uridylating *MYB33* 5′-cleavage fragments, as shown here using four independent biological replicates in the Col-0 genetic background. In addition, and as previously observed (Ren et al., 2014), the size of U-tails detected in *heso1-1* was reduced as compared to WT, with mainly short U-tails (<2 Us) detected in *heso1-1* (**Figure 3**).

**FIGURE 4:**
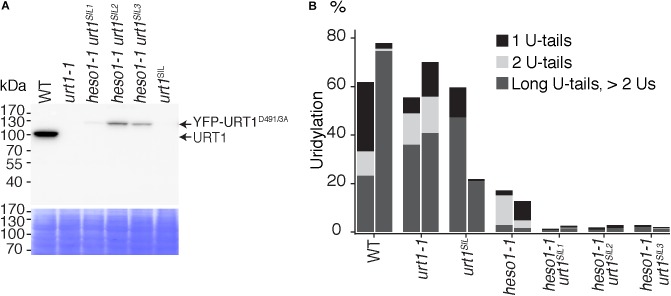
Respective contributions of HESO1 and URT1 in the uridylation of *MYB33* RISC 5′-cleavage fragments. **(A)** Western blot analysis of WT, *urt1-1* T-DNA mutant, co-suppressed *urt1* mutant (*urt1^SIL^*), and three double mutant lines *heso1-1 urt1^SIL^*. Molecular weight markers are indicated in kDa. A portion of the membrane stained with Coomassie blue is shown as loading control. Please note that the construct used to co-suppress *URT1* in the *heso1-1* background expresses an inactive version of URT1 fused to YFP. Uncropped images are shown in **Supplementary Figure S1**. **(B)** Percentages of uridylated *MYB33* RISC 5′-cleavage fragments in two biological replicates for WT, *urt1-1*, the *urt1^SIL^* line, *heso1-1*, and the three *heso1-1 urt1^SIL^* lines (i.e., six *heso1-1 urt1^SIL^* samples). Percentages of long (>2 Us), 2 U- and 1 U-tails are indicated by dark gray, light gray, and black, respectively.

The residual uridylation in *heso1-1* indicates the involvement of an alternative TUTase. A good candidate for this activity is URT1, the second TUTase that has been identified in Arabidopsis (Sement et al., 2013). To date, the possible involvement of URT1 in the uridylation of 5′ RISC-cleaved mRNAs, including *MYB33*, has been proposed but not tested experimentally. Testing this hypothesis requires the production of a *heso1 urt1* double mutant. To this end, we crossed the *heso1-1* and *urt1-1* single mutants. However, we failed to recover the expected double mutant in the F2 progeny. This failure is yet unexplained but we could obtain lines that were originally designed to overexpress an inactive version of URT1, but that in fact co-suppress the endogenous *URT1* gene in the *heso1-1* background. We selected three *heso1-1* lines for which the endogenous URT1 was not detected anymore by western blot analysis, revealing a drastic downregulation of URT1 (**Figure 4A**). These lines, which have no particular phenotype when grown under optimal conditions, are called *heso1-1 urt1^SIL1^, heso1-1 urt1^SIL2^*, and *heso1-1 urt1^SIL3^* thereafter. The uridylation of *MYB33* 5′-cleavage fragments was down to background levels in both biological replicates for three *heso1-1 urt1^SIL^* lines as compared with the single *heso1-1* mutant (**Figure 4B**). Therefore, both HESO1 and URT1 participate in uridylating *MYB33* 5′-cleavage fragments, albeit HESO1 is clearly the main TUTase involved in uridylating these fragments.

Of note, HESO1 and URT1 might have a distinct contribution in the uridylation of *MYB33* 5′-cleavage fragments. HESO1 can synthesize short and long U-extensions, but URT1 seems to add only one or two uridines (**Figure 4B**). Interestingly, a similar distinction was proposed for HESO1 and URT1 in uridylating small RNAs. URT1 was proposed to add a single uridine to small RNAs to favor the subsequent action of HESO1, which prefers 3′ extremities ending with uridines (Tu et al., 2015; Yu et al., 2017). A comparable scenario could exist for RISC 5′-cleavage fragments although additional investigation is required to confirm this hypothesis. In any case, and as previously observed for small RNAs, uridine addition by URT1 to RISC 5′-cleavage fragments does not seem to be a prerequisite to the action of HESO1, at least for *MYB33* 5′-cleavage fragments.

### Respective Contribution of HESO1 and URT1 in the Accumulation of *MYB33* 5′-Cleavage Fragments

**FIGURE 5:**
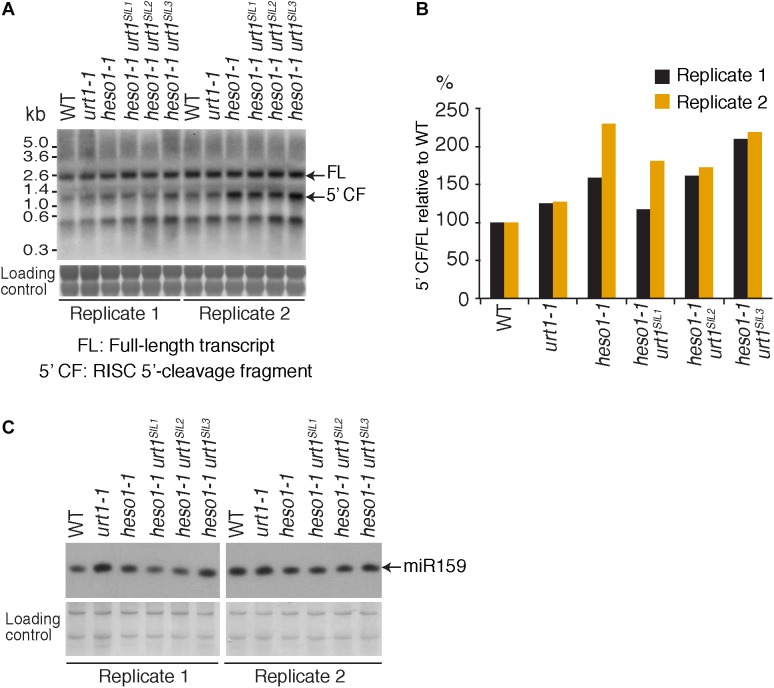
*MYB33* 5′-cleavage fragments accumulate in *heso1-1* **(A)** Northern blot analysis of WT, *urt1-1*, and *heso1-1* mutant lines using a probe 5′ of the cleavage site to detect *MYB33* full-length mRNA and the RISC 5′-cleavage fragment. Molecular weight markers are indicated in kb. A portion of the membrane stained with methylene blue is shown as loading control. Uncropped images are shown in **Supplementary Figure S2**. **(B)** *MYB33* RISC 5′-cleavage fragments accumulate mostly in the absence of HESO1. The accumulation of *MYB33* RISC 5′-cleavage fragments (5′ CF) relative to its full-length (FL) mRNA was determined by integrating the signals in **B** with a Phosphorimager. 5′ CF/FL values for each lane were normalized to the 5′ CF/FL ratio obtained for the WT control of each replicate. Two replicates are shown in black and orange, respectively, for WT, *urt1-1*, the *urt1^SIL^* line, *heso1-1*, and the three *heso1-1 urt1^SIL^* lines (i.e., six *heso1-1 urt1^SIL^* samples). **(C)** Northern blot analysis of WT, *urt1-1*, and *heso1-1* mutant lines using a probe to detect *miR159*. A portion of the membrane stained with methylene blue is shown as loading control. Uncropped images are shown in **Supplementary Figure S2**.

To further check the predominant role of HESO1 in the metabolism of *MYB33* 5′-cleavage fragments, we analyzed their accumulation by northern blot analysis and phosphorimager quantification (**Figure 5A**). The accumulation of *MYB33* RISC 5′-cleavage fragments in each sample was calculated relative to its full-length mRNA and each ratio was normalized to the ratio obtained for the WT control for each of the two replicates. As previously observed (Ren et al., 2014), *MYB33* 5′-cleavage fragments accumulated to higher levels in *heso1-1* with respect to WT (**Figure 5B**), although for unknown reasons the accumulation seemed variable in both replicates. Yet, our northern analysis confirmed that uridylation by HESO1 likely destabilizes *MYB33* 5′-cleavage fragments. The single *urt1* mutation seemed to have no major effect on this accumulation. Furthermore, *MYB33* 5′-cleavage fragments accumulated to similar levels in the *heso1-1 urt1^SIL^* lines as compared to the single *heso1-1* mutant (**Figure 5**). In other words, there was no additive effect of the lack of URT1 and HESO1, and this observation points to HESO1 as the main TUTase controlling the accumulation of *MYB33* 5′-cleavage fragments. Of note, miR159 accumulated to similar levels when HESO1 is absent, ruling out a higher rate of production of *MYB33* 5′-cleavage fragments in *heso1-1* mutants (**Figure 5C**). Altogether, the 3′ RACE-seq and northern analyses indicate that HESO1 is the main TUTase modifying *MYB33* 5′-cleavage fragments. Although URT1 could add short uridine extensions to *MYB33* 5′-cleavage fragments, it does not appear to be a limiting factor neither in the uridylation nor in the destabilization of this fragment produced by RISC cleavage.

### mRNA 5′ Fragments Are Nibbled at RISC Cleavage Site in the Absence of Uridylation

**FIGURE 6:**
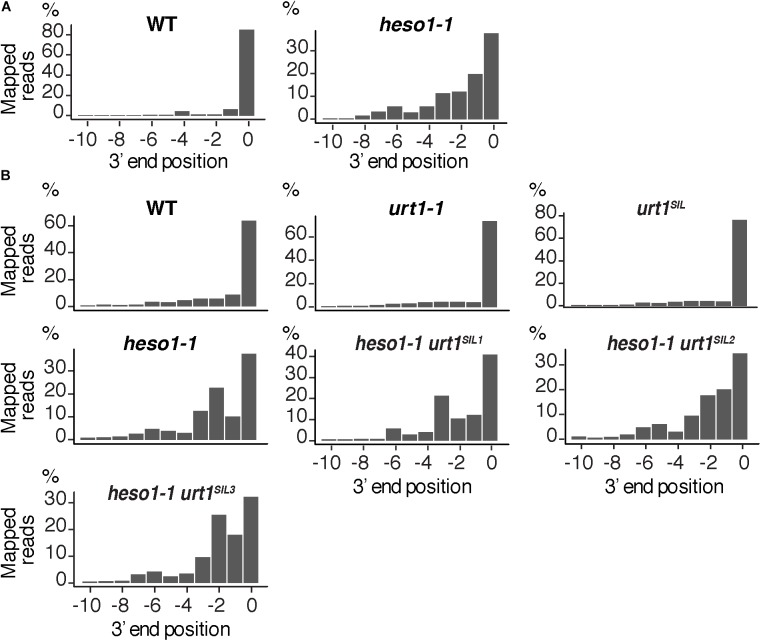
Nibbled *MYB33* RISC 5′-cleavage fragments accumulate in the absence of HESO1. Positions of 3′ extremities of *MYB33* RISC 5′-cleavage fragments mapped in a –10/0 window for **(A)** four biological replicates, WT and *heso1-1*, or **(B)** two biological replicates for WT, *urt1-1*, the *urt1^SIL^* line, *heso1-1*, and the three *heso1-1 urt1^SIL^* lines. Graphs are shown separately for each of the replicates in **Supplementary Figures S3, S4**.

The 3′ truncation up to several hundreds of nucleotides upstream of the RISC cleavage site was previously observed for *MYB33* 5′-cleavage fragments in the *heso1-2* mutant (Ren et al., 2014). We took advantage of the depth of the 3′ RACE-seq procedure to analyze at high resolution the 3′ extremities of *MYB33* 5′-cleavage fragments in the vicinity of the cleavage site. Although the vast majority of extremities in the four WT biological replicates mapped at position 0, different patterns were observed for *heso1-1*. The patterns were not completely identical in the four biological replicates, but they all revealed the same trend: the 3′ extremities were spread over positions from -10 to 0 (**Figure 6** and **Supplementary Figure S3**). This observation reveals that the *MYB33* 5′-cleavage fragments that accumulate in the absence of HESO1 are nibbled at close proximity to the cleavage site. This nibbling shortens *MYB33* 5′-cleavage fragments by up to 8–9 nucleotides (**Figure 6A** and **Supplementary Figure S3**). Such a nibbling was not observed in the single *urt1-1* mutant (**Figure 6B** and **Supplementary Figure S4**) but it was consistently observed in *heso1-1* and not aggravated in *heso1-1 urt1^SIL^* mutants (**Figure 6B** and **Supplementary Figure S4**). Therefore, the nibbling is solely attributed to the absence of HESO1, but not of URT1, in the case of *MYB33* 5′-cleavage fragments.

**FIGURE 7:**
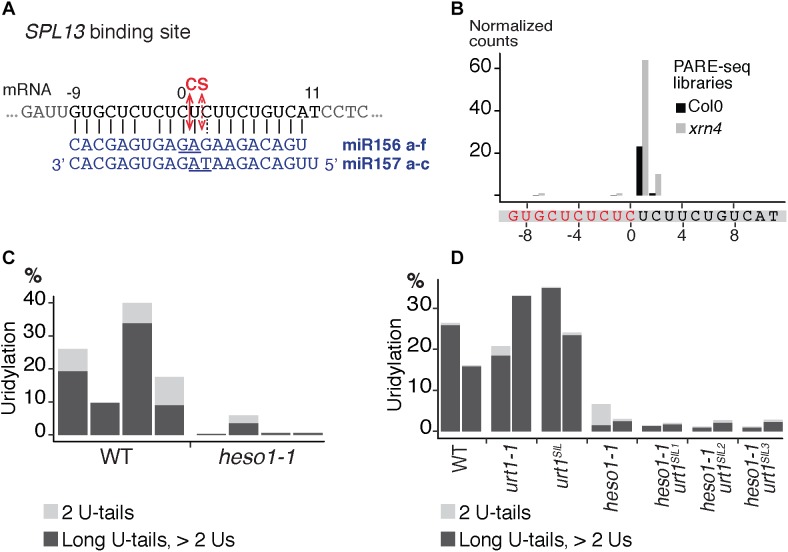
Respective contributions of HESO1 and URT1 in the uridylation of *SPL13* RISC 5′-cleavage fragments. **(A)** Schematic representation of the cleavage of *SPL13* mRNA by AGO1 loaded with miR156 or miR157. The cleavage sites (CS) are predicted between the positions 0 and +1 or between position +1 and +2 for miR156 and miR157, respectively, and are defined by the 10th and 11th nucleotides (underlined) of the miRNAs. **(B)** 5′ end mapping using PARE-seq data (German et al., 2008) of RISC 3′-cleavage fragments in WT and *xrn4*. **(C,D)** Percentages of uridylated *SPL13* RISC 5′-cleavage fragments in **(C)** four biological replicates for WT and *heso1-1* or **(D)** two biological replicates for WT, *urt1-1*, the *urt1^SIL^* line, *heso1-1*, and the three *heso1-1 urt1^SIL^* lines (i.e., six *heso1-1 urt1^SIL^* samples). Percentages of long (>2 Us) and 2 U-tails are indicated by dark gray and light gray, respectively.

**FIGURE 8:**
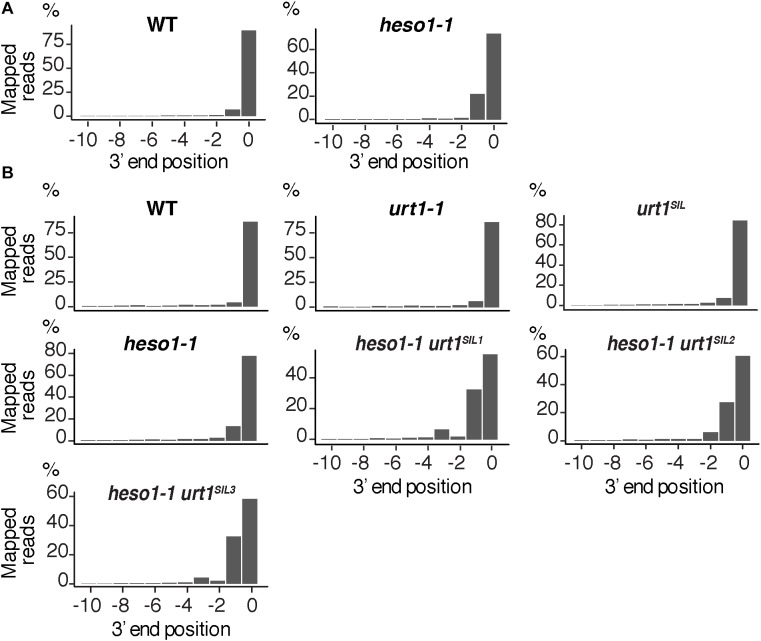
Nibbled *SPL13* RISC 5′-cleavage fragments accumulate in the absence of uridylation. Positions of 3′ extremities of *SPL13* RISC 5′-cleavage fragments mapped in a –10/0 window for **(A)** four biological replicates, WT and *heso1-1*, or **(B)** two biological replicates for WT, *urt1-1*, the *urt1^SIL^* line, *heso1-1*, and the three *heso1-1 urt1^SIL^* lines. Graphs are shown separately for each of the replicates in **Supplementary Figures S5, S6**.

We then analyzed the respective contribution of HESO1 and URT1 in uridylating the 5′ fragments produced by RISC cleavage of *Squamosa promoter-binding-like protein 13* (*SPL13*) mRNAs that are targets of miR156 and miR157 (**Figure 7A**). PARE-seq data identify a major and a minor 5′ extremity for the 3′ fragments produced by RISC cleavage (**Figure 7B**). Therefore, it is possible that in addition to the major cleavage site denoted 0 in **Figure 7A**, a minor site exists at position +1. This minor site at +1 presumably results from the action of miR157 (**Figure 7A**; He et al., 2018). Because nucleotide +1 is a U, it is not possible to determine in the 3′ RACE-seq data whether this U is encoded or added post-transcriptionally. To eliminate this uncertainty that could affect the proportion of uridylated versus non-uridylated fragments, we considered only tails of at least two nucleotides. Of note, not considering the 1 U extensions may lead to the underestimation of the action of URT1 and/or HESO1 in adding 1 U. The overall level of uridylation of *SPL13* 5′-cleavage fragments was lower than for *MYB33*, with percentage of uridylation below 40% and an increased variability between replicates (**Figures 7C,D** and **Supplementary Table S4**). Yet, a similar pattern was observed for both targets: uridylation of RISC 5′-cleavage fragments is mostly reduced in the absence of HESO1 and close to background levels in *heso1-1 urt1^SIL^* lines (**Figures 7C,D**). Interestingly, the nibbling of RISC 5′-cleavage fragments was increased in the six replicates of *heso1-1 urt1^SIL^* although to a lesser extent than the one observed for *MYB33* (**Figure 8**). This observation confirms the accumulation of RISC 5′-cleavage fragments that are nibbled close to the cleavage site in case of defective uridylation. The greater accumulation of nibbled fragments in absence of HESO1 and URT1 suggests that in the case of *SPL13* 5′-cleavage fragments, the absence of uridylation *per se* is responsible for this accumulation.

Two, non-mutually exclusive, interpretations can explain the accumulation of nibbled RISC 5′-cleavage fragments in the absence of uridylation. First, uridylation of the nibbled fragments could trigger their degradation. Their fast turn-over would explain that they are not detected in WT plants. However, those fragments would accumulate in the absence of the TUTases. The second alternative possibility would be that in the presence of HESO1 and/or URT1, the 3′ extremities are not accessible to the activity, presumably a 3′-5′ exoribonucleolytic activity, that generates the nibbled RNA species. Such a possibility was previously evoked to explain the accumulation of truncated 5′-cleavage fragments in the *heso1-2* mutant (Ren et al., 2014). Solving this question entails the identification of all ribonucleases involved in the metabolism of 5′ RISC-cleaved transcripts.

## Conclusion

Here, we report the respective contribution of HESO1 and URT1 in the metabolism of two 5′ RISC-cleaved mRNAs. In addition, we show the applicability of 3′ RACE-seq to map the 3′ ends of 5′ RISC-cleaved transcripts and to identify untemplated nucleotides added at these 3′ ends. The depth of 3′ RACE-seq will be useful for both qualitative and quantitative comparisons across different targets, tissues, conditions or genotypes. For instance, different RISC 5′-cleavage fragments could be investigated to identify both common and specific behaviors of these RNA fragments produced by post-transcriptional gene silencing. Also, the full machinery involved in the degradation of RISC 5′-cleavage fragments needs to be characterized. This is an on-going process with the recent identification of RICE exoribonucleases (Zhang et al., 2017) or the recent description that components of the NSD pathway and the Ski complex, a major co-factor of the cytosolic RNA exosome, are involved in the degradation of RISC 5′-cleavage fragments (Branscheid et al., 2015; Szádeczky-Kardoss et al., 2018). Yet the direct involvement of the RNA exosome in the clearance of RISC 5′-cleavage fragments remains to be demonstrated in Arabidopsis. The impact of SUPPRESSOR OF VARICOSE (SOV), whose ortholog is called Dis3L2 in non-plant eukaryotes, on the degradation of RISC 5′-cleavage fragments could also be investigated. Dis3L2 is a 3′-5′ exoribonuclease belonging to the RNase II family and whose activity is stimulated by uridylation in fission yeast, fruit fly or human cells (De Almeida et al., 2018). Whether SOV participates in the clearance of uridylated 5′ fragments of RISC-cleaved transcripts could be reliably addressed using 3′ RACE-seq and by comparing Col-0 and *Ler* accessions, because a point mutation affects SOV activity in Col-0 (Zhang et al., 2010). All these examples illustrate that a large number of samples must be analyzed with sufficient depth and replicates to draw reliable conclusions. The 3′ RACE-seq method adapted to the analysis of RISC 5′-cleavage fragments will contribute to fully characterize the tailing and nibbling events linked to the metabolism of these fragments and to address the respective roles of distinct factors of the RNA degradation machinery in this process.

## Author Contributions

DG and HZ conceived and designed the study, wrote the paper, and acquired funding. HZ, A-CJ, and HS performed the experiments. HZ performed the bioinformatics analysis. A-CJ and HS edited the manuscript. HZ and HS prepared the illustrations.

## Conflict of Interest Statement

The authors declare that the research was conducted in the absence of any commercial or financial relationships that could be construed as a potential conflict of interest.

## Supplementary Material

FIGURE S1 related to Figure 4 Uncropped images of the western blot analysis and the membrane stained with Coomassie blue shown in **Figure 4**.

FIGURE S2 related to Figure 5 Uncropped images of the northern blot analysis and the membrane stained with methylene blue for **(A)** *MYB33* 5′ fragment analysis and for **(B)** miR159 analysis.

FIGURE S3 related to Figure 6 Nibbled *MYB33* RISC 5′-cleavage fragments accumulate in the absence of HESO1. Positions of 3′ extremities of *MYB33* RISC 5′-cleavage fragments mapped in a -10/0 window for four biological replicates in WT and *heso1-1*. Graphs are shown separately for each of the four replicates.

FIGURE S4 related to Figure 6 Nibbled *MYB33* RISC 5′-cleavage fragments accumulate in the absence of HESO1. Positions of 3′ extremities of *MYB33* RISC 5′-cleavage fragments mapped in a -10/0 window for two biological replicates for WT, *urt1-1*, the *urt1^SIL^* line, *heso1-1*, and the three *heso1-1 urt1^SIL^* lines. Graphs are shown separately for each of the two replicates.

FIGURE S5 related to Figure 8 Positions of 3′ extremities of *SPL13* 5′-cleavage fragments mapped in a -10/0 window for four biological replicates in WT and *heso1-1*. Graphs are shown separately for each of the four replicates.

FIGURE S6 related to Figure 8 Nibbled *SPL13* RISC 5′-cleavage fragments accumulate in the absence of HESO1 and URT1. Positions of 3′ extremities of *SPL13* RISC 5′-cleavage fragments mapped in a -10/0 window for two biological replicates for WT, *urt1-1*, the *urt1^SIL^* line, *heso1-1*, and the three *heso1-1 urt1^SIL^* lines. Graphs are shown separately for each of the two replicates.

TABLE S1 List of primers used in this study.

TABLE S2 Summary of the number of reads analyzed at each step of the data processing for each 3′ RACE-seq library.

TABLE S3 Exhaustive list of extensions found by 3′ RACE-seq for *MYB33* 5′-cleavage fragments in WT for four biological replicates from dataset #1.

TABLE S4 Exhaustive list of extensions found by 3′ RACE-seq for *SPL13* 5′-cleavage fragments in WT for four biological replicates from dataset #1.

DATA SHEET S1 Scripts for 3′ RACE-seq data processing, related to the analysis of 5′ mRNA fragments generated by RISC cleavage of *MYB33* mRNAs.

DATA SHEET S2 Scripts for 3′ RACE-seq data processing, related to the analysis of 5′ mRNA fragments generated by RISC cleavage of *SPL13* mRNAs.
