# Supplementary material for: Respective Contributions of URT1 and HESO1 to the Uridylation of 5′ Fragments Produced From RISC-Cleaved mRNAs
Source: Front Plant Sci. 2018 Oct 9;9:1438. doi: 10.3389/fpls.2018.01438 (PMC6191825; doi:10.3389/fpls.2018.01438)
Supplement: FIGURE S1 related to Figure 4 — Uncropped images of the western blot analysis and the membrane stained with Coomassie blue shown in Figure 4. [file Image_1.pdf]

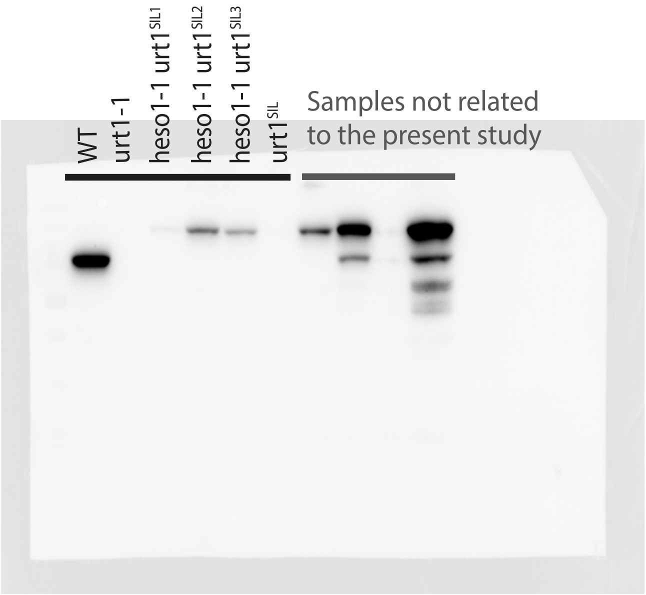

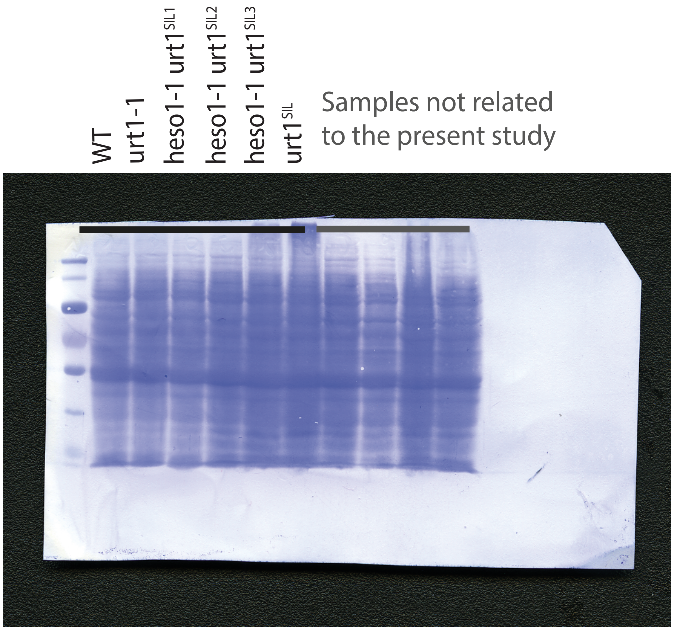

Supplementary Figure 1, related to Figure 4. Uncropped images of the western blot analysis and the membrane stained with Coomassie blue.
