# Supplementary material for: Respective Contributions of URT1 and HESO1 to the Uridylation of 5′ Fragments Produced From RISC-Cleaved mRNAs
Source: Front Plant Sci. 2018 Oct 9;9:1438. doi: 10.3389/fpls.2018.01438 (PMC6191825; doi:10.3389/fpls.2018.01438)
Supplement: FIGURE S2 related to Figure 5 — Uncropped images of the northern blot analysis and the membrane stained with methylene blue for (A) MYB33 5′ fragment analysis and for (B) miR159 analysis. [file Image_2.pdf]

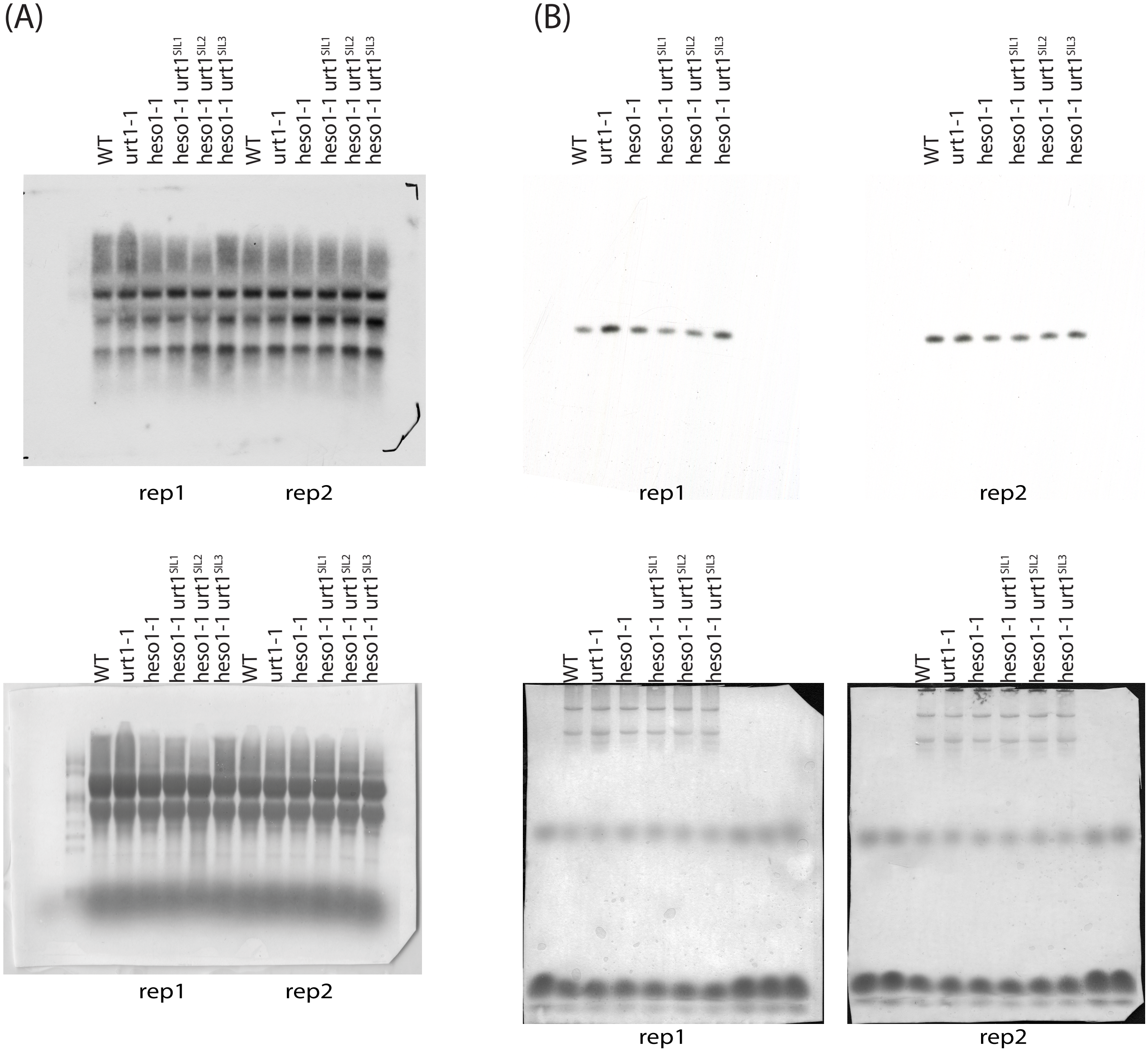

Supplementary Figure 2, related to Figure 5. Uncropped images of the northern blot analysis and the membrane stained with methylene blue for (A) MYB33 5′ fragment analysis and for (B) miR159 analysis
