# Supplementary material for: Respective Contributions of URT1 and HESO1 to the Uridylation of 5′ Fragments Produced From RISC-Cleaved mRNAs
Source: Front Plant Sci. 2018 Oct 9;9:1438. doi: 10.3389/fpls.2018.01438 (PMC6191825; doi:10.3389/fpls.2018.01438)
Supplement: FIGURE S3 related to Figure 6 — Nibbled MYB33 RISC 5′-cleavage fragments accumulate in the absence of HESO1. Positions of 3′ extremities of MYB33 RISC 5′-cleavage fragments mapped in a -10/0 window for four biological replicates in WT and heso1-1. Graphs are shown separately for each of the four replicates. [file Image_3.pdf]

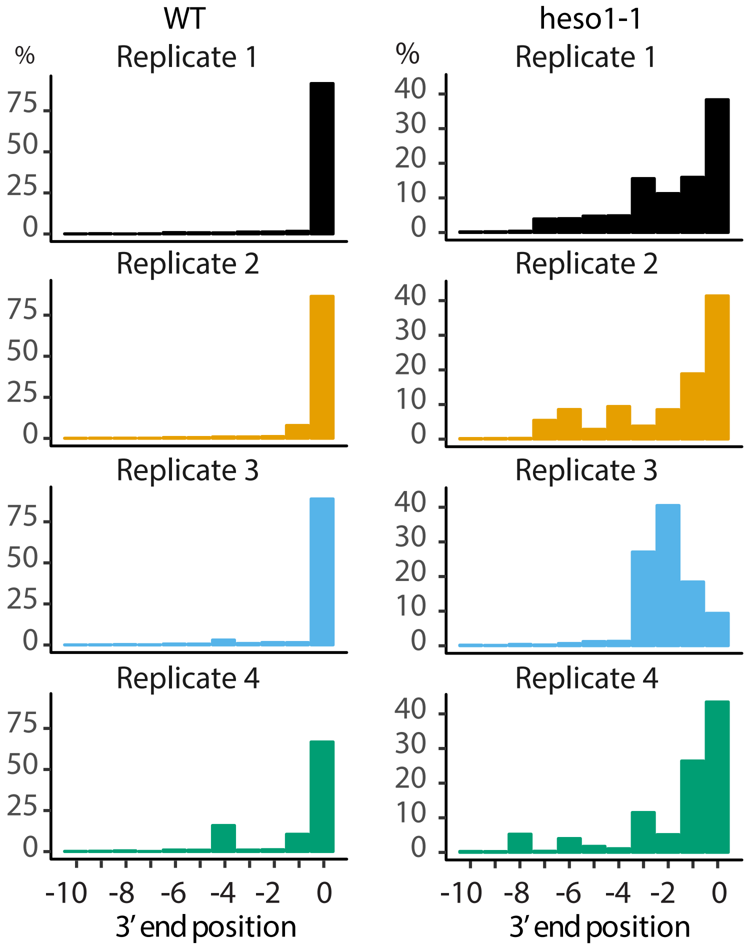

Supplementary Figure 3, related to Figure 6. Nibbled MYB33 5′-cleavage fragments accumulate in the absence of HESO1. Positions of 3′ extremities of MYB33 5′-cleavage fragments mapped in a -10/0 window for four biological replicates in WT and heso1-1. Graphs are shown separately for each of the four replicates.
