# Supplementary material for: Respective Contributions of URT1 and HESO1 to the Uridylation of 5′ Fragments Produced From RISC-Cleaved mRNAs
Source: Front Plant Sci. 2018 Oct 9;9:1438. doi: 10.3389/fpls.2018.01438 (PMC6191825; doi:10.3389/fpls.2018.01438)
Supplement: FIGURE S5 related to Figure 8 — Positions of 3′ extremities of SPL13 5′-cleavage fragments mapped in a -10/0 window for four biological replicates in WT and heso1-1. Graphs are shown separately for each of the four replicates. [file Image_5.pdf]

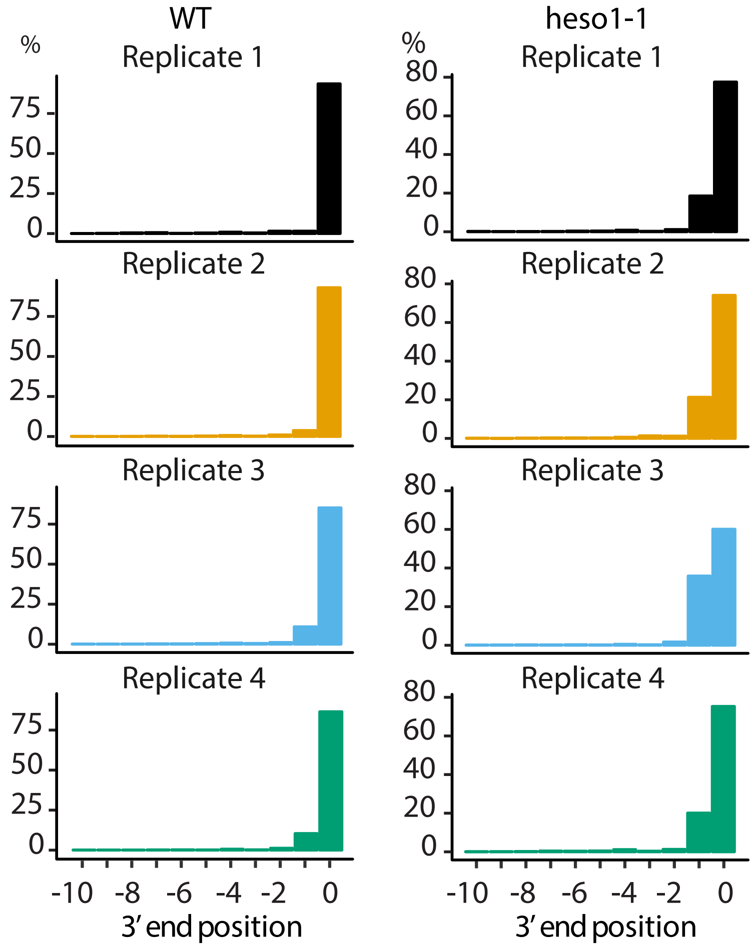

Supplementary Figure 5, related to Figure 8. Positions of 3′ extremities of SPL13 5′-cleavage fragments mapped in a -10/0 window for four biological replicates in WT and heso1-1. Graphs are shown separately for each of the four replicates.
