# Supplementary material for: Respective Contributions of URT1 and HESO1 to the Uridylation of 5′ Fragments Produced From RISC-Cleaved mRNAs
Source: Front Plant Sci. 2018 Oct 9;9:1438. doi: 10.3389/fpls.2018.01438 (PMC6191825; doi:10.3389/fpls.2018.01438)
Supplement: FIGURE S6 related to Figure 8 — Nibbled SPL13 RISC 5′-cleavage fragments accumulate in the absence of HESO1 and URT1. Positions of 3′ extremities of SPL13 RISC 5′-cleavage fragments mapped in a -10/0 window for two biological replicates for WT, urt1-1, the urt1SIL line, heso1-1, and the three heso1-1 urt1SIL lines. Graphs are shown separately for each of the two replicates. [file Image_6.pdf]

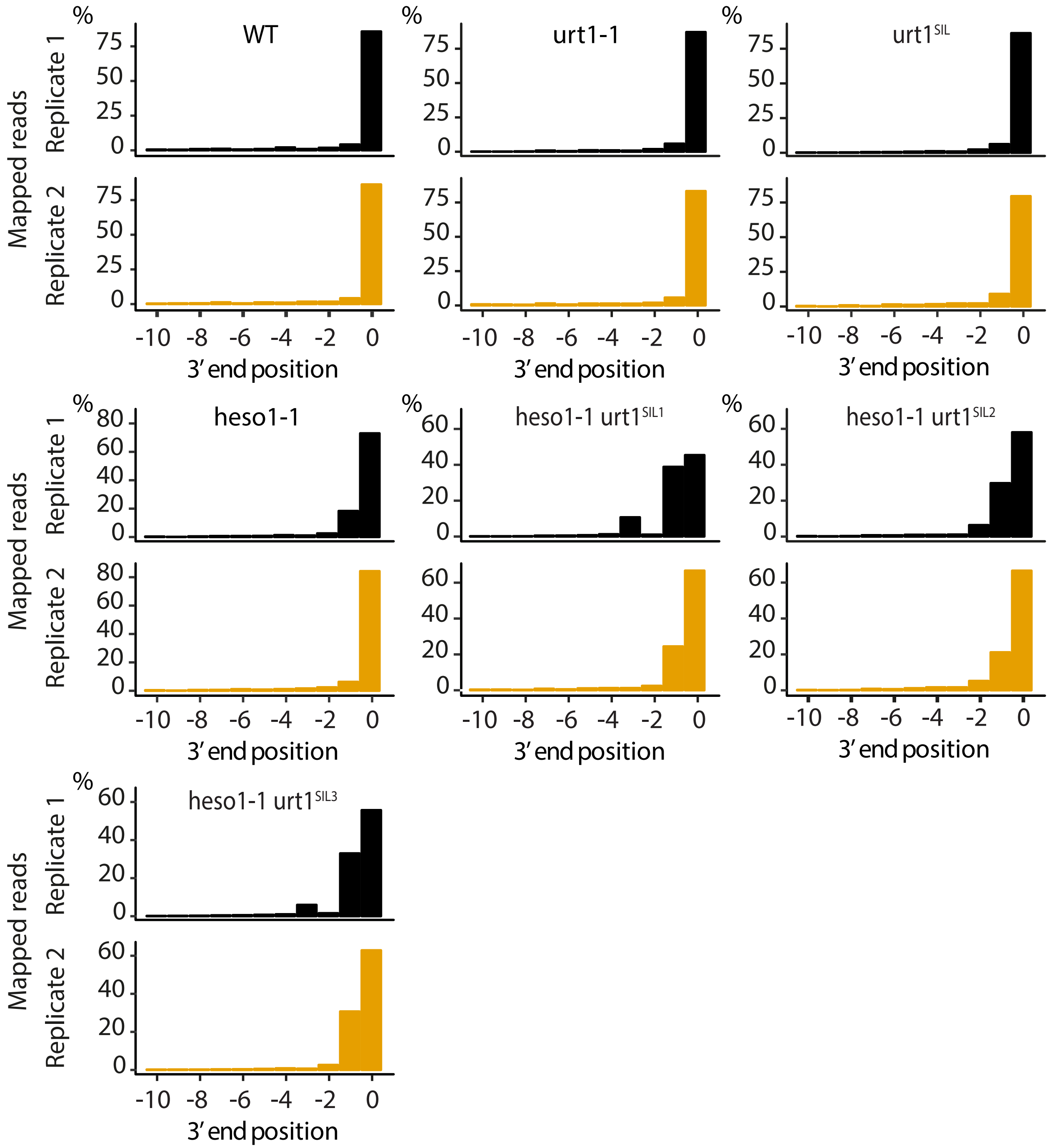

Supplementary Figure 6, related to Figure 8. Nibbled SPL13 5′-cleavage fragments accumulate in the absence of HESO1 and URT1. Positions of 3′ extremities of SPL13 5′-cleavage fragments mapped in a -10/0 window for two biological replicates in WT, urt1-1, urt1[SIL], heso1-1 and each of the three heso1-1 urt1[SIL] lines. Graphs are shown separately for each of the two replicates.
